# Characterization of Multisugar-Binding C-Type Lectin (*Spli*Lec) from a Bacterial-Challenged Cotton Leafworm, *Spodoptera littoralis*

**DOI:** 10.1371/journal.pone.0042795

**Published:** 2012-08-20

**Authors:** AlaaEddeen M. Seufi, Fatma H. Galal, Elsayed E. Hafez

**Affiliations:** 1 Department of Entomology, Faculty of Science, Cairo University, Giza, Egypt; 2 Department of Plant Protection and Molecular Diagnosis, ALCADRI, City for Scientific Research and Technology Application, New Borg ElArab, Alex, Egypt; Ghent University, Belgium

## Abstract

**Background:**

Various proteins that display carbohydrate-binding activity in a Ca^2+^-dependent manner are classified into the C-type lectin family. They have one or two C-type carbohydrate-recognition domains (CRDs) composed of 110–130 amino acid residues in common. C-type lectins mediate cell adhesion, non-self recognition, and immuno-protection processes in immune responses and thus play significant roles in clearance of invaders, either as cell surface receptors for microbial carbohydrates or as soluble proteins existing in tissue fluids. The lectin of *Spodoptera littoralis* is still uncharacterized.

**Methodology:**

A single *orf* encoding a deduced polypeptide consisting of an 18-residue signal peptide and a 291-residue mature peptide, termed *Spli*Lec, was isolated from the haemolymph of the cotton leafworm, *S. littoralis*, after bacterial challenge using RACE-PCR. Sequence analyses of the data revealed that *Spli*Lec consists of two CRDs. Short-form CRD_1_ and long-form CRD_2_ are stabilized by two and three highly conserved disulfide bonds, respectively. *Spli*Lec shares homology with some dipteran lectins suggesting possible common ancestor. The purified *Spli*Lec exhibited a 140-kDa molecular mass with a subunit molecular mass of 35 kDa. The hemagglutination assays of the *Spli*Lec confirmed a thermally stable, multisugar-binding C-type lectin that binds different erythrocytes. The purified *Spli*Lec agglutinated microorganisms and exhibited comparable antimicrobial activity against gram (+) and gram (−) bacteria too.

**Conclusions:**

Our results suggested an important role of the *Spli*Lec gene in cell adhesion and non-self recognition. It may cooperate with other AMPs in clearance of invaders of *Spodoptera littoralis*.

## Introduction

After pathogens penetrate the insects' structural barriers, they rely solely on an efficient innate immune system which shares many characteristics with the innate immune system of vertebrates. Insect innate immune system comprises both humoral and cellular responses [1], [2]. Insect humoral defenses include the production of a potent arsenal of antimicrobial peptides (AMPs) [1], [2], coagulation, and melanization led by protease cascades [3]. Insect cellular defense refers to haemocyte-mediated immune responses, such as phagocytosis, nodulation, and encapsulation [4]. The encapsulation process involves cell adhesion and melanization [5]. Lectins are an important class of carbohydrate-binding proteins that have several distinct biological activities. They mediate cell adhesion (i.e. bind to microbial surface components), non-self recognition and immuno-protection processes in immune responses [6]. They exist in a wide variety of plants, animals, fungi, bacteria and viruses [7] and play significant role in clearance of invaders, either as cell surface receptors for microbial carbohydrates or as soluble proteins existing in tissue fluids [8]. Such proteins are known as pattern recognition receptors (PRPs), because they bind to the pathogen associated molecular patterns (PAMPs) present in the array of carbohydrate components on the surface of microorganisms and consequently, trigger a series of protective immune responses [9]. Various proteins that display carbohydrate-binding activity in a calcium-dependent manner are classified into the C-type lectin family [10]. They contain C-type carbohydrate-recognition domains (CRDs) or C-type lectin domains (CTLDs) composed of 110–130 amino acid residues in common. These CRDs or CTLDs contain a characteristic double-loop (loop in a loop) stabilized by two or three highly conserved disulfide bonds. The vertebrate C-type lectins are usually multi-domain lectins and they fall into seven groups (I–VII) [11]. Seven new groups (VIII–XIV) were added in the revised classification in 2002 [12] and three new groups (XV–XVII) were updated, recently [13]. In contrast, the invertebrate C-type lectins are mostly single-domain proteins, but C-type lectins that contain two CRDs are characterized too. Although all C-type lectin CRDs have sequence similarity, they can be divided into two types: a “short form” approximately 115 residues long and a “long form” approximately 130 residues long, which includes two additional disulfide-bonded cysteine residues at the amino terminus [10], [11]. In recent years, more and more C-type lectins with two tandem CRDs have been identified and characterized from invertebrates, especially from insects [14]–[16]. Examples of the C-type lectins with two tandem CRDs include the *M. sexta* immunolectins (IML-1, IML-2, IML-3 and IML-4) which serve as humoral PRPs [3], LPS-binding lectins from the silkworm, *Bombyx mori* [17] and the fall webworm, *Hyphantria cunea* [18].

In this paper, the full length cDNA of a multisugar-binding C-type lectin with two tandem CRDs from *S. littoralis*, *Spli*Lec, was isolated. Sequence characterization, phylogenetic analysis, hemagglutinating activity, carbohydrate-binding specificity, microbial agglutination and antimicrobial activities were investigated for the immunized haemolymph and the purified *Spli*Lec, as well.

## Materials and Methods

### Insects

Laboratory colony of the cotton leafworm, *S. littoralis*, used for our experiments was originally collected from a private okra field at Giza, Egypt in 1995 and maintained in the insectary of the Department of Entomology, Faculty of Science, Cairo University according to the technique described by El-Defrawi *et al.* [19]. Larvae were reared on a semisynthetic diet described by Levinson and Navon [20] and kept at 25°C, 65–70% RH and 14L∶ 10D photoperiod cycle. All necessary permits for the described field studies were obtained from the owner of the private land. These field studies did not involve endangered or protected species.

### Bacterial strains

Two gram (+) bacteria, *Staphylococcus aureus* and *Streptococcus sanguinis* and three gram (−) bacteria, *Escherichia coli* (D_31_), *Proteus vulgaris* and *Klebsiella pneumoniae* were obtained from the Unit for Genetic Engineering and Agricultural Biotechnology, Faculty of Agriculture, Ain Shams University and used for insect immunization. Bacteria were grown in a peptone medium (1%), supplemented with 1% meat extract and 0.5% NaCl, at 37°C in a rotary shaker.

### Insect immunization and haemolymph collection

Insect immunization was performed by injecting 20 newly moulted fourth instar larvae with 2–5 µl of approximately 1×10^6^ (cells/ml) log phase bacteria dissolved in membrane-filtered saline using a thin-needled microsyringe. Haemolymph was collected 1, 6, 12, 24, 48 and 72 h post-infection (p.i.) at 4°C (500 µl/each), containing few crystals of phenylthiourea to prevent melanization. Haemolymph was pooled by piercing a proleg with a fine, sterile needle. Haemolymph was aliquoted (100 µl each) and stored at −80°C for a weak until investigated. The same procedures were applied to control group except it was injected with saline without bacteria.

### RNA extraction and reverse transcription

Total RNA of insect haemolymph (300–500 µl) was extracted using RNeasy kit according to manufacturer's instructions (Qiagen, Germany). Residual genomic DNA was removed using RNase-free DNase (Ambion, Germany). RNA was dissolved in DEPC-treated water, quantified using a BioPhotometer 6131 (Eppendorf) and analyzed on 1.2% formaldehyde agarose gel to ensure its integrity. The 260/280 and 260/230 ratios were examined for protein and solvent contamination. A total of 100 ng of DNA-free total RNA was converted into cDNA using a mix of random and oligodT20 primers according to the ABgene protocol (ABgene, Germany). Synthesis of the first cDNA strand was performed in a thermal cycler (Eppendorf, Mastercycler 384, Germany) programmed at 42°C for 1 h, 72°C for 10 min and a soak at 4°C. cDNA was aliquoted and stored at −80°C untill processed (within a weak).

### Differential display using primers corresponding to lectin sequence (DD-PCR)

A total reaction volume of 25 µl containing 2.5 µl PCR buffer, 1.5 mM MgCl_2_, 200 µM dNTPs, 1 U *Taq* DNA polymerase (AmpliTaq, Perkin-Elmer), 2.5 µl of 10 pmol/µl primer (Table S1) and 2.5 µl of each cDNA was cycled in a DNA thermal cycler (Eppendorf, Mastercycler 384, Germany). The amplification program was one cycle at 94°C for 5 min (hot start), followed by 40 cycles at 94°C for 1 min, 40°C for 1 min and 72°C for 1 min. The reaction was then incubated at 72°C for 10 min for final extension. PCR product was visualized on 1.5% agarose gel and photographed using gel documentation system. For DNA contamination assessment, a no-reverse transcription control reaction was performed.

### Primer design and RT-PCR

Five reproducible bacterial-induced bands were eluted, cloned into *PCR-TOPO* vector (Invitrogen, USA) and sequenced using M_13_ universal primer. Sequencing was performed using T^7^Sequencing™ kit (Pharmacia, Biotech, USA) and model 310 automated sequencer (Applied Biosystems, Foster City, CA, USA). Analyses of nucleotide and deduced amino acid sequences was carried out using EditSeq-DNAstar Inc., Expert Sequence Analysis software, Windows 32 Edit Seq 4.00 (1989–1999) and ExPasy database (http://expasy.org/tools/dna.html). Blast search for alignment of the obtained sequence with the published ones was done using database of NCBI (http://blast.ncbi.nlm.nih.gov/Blast.cgi).

Based on the sequence and alignment data, specific primers (LecSF_1,2_ and LecSR_1,2_) for lectin-related sequences were designed (Table S1) and tried for reverse transcription polymerase chain reaction (RT-PCR). Primers were designed by the rules of highest maximum efficiency and sensitivity rules were followed to avoid formation of self and hetero-dimers, hairpins and self-complementarity. RT-PCR reaction was performed as previously described in this section regarding to the optimum annealing temperature (T_a_) for each specific primer set. Positive PCR products were visualized and eluted from the gel using GenClean Kit (Invitrogen Corporation, San Diego, CA, USA) following the manufacturer's instructions. The purified PCR product (*Spli*Lec) was cloned into *PCR-TOPO* vector with TOPO TA cloning kit (Invitrogen, USA) following the manufacturer's instructions. Ligation mix was used to transform competent *E. coli* strain TOPO_10_ provided with the cloning kit. White colonies were screened using PCR as described earlier in this section. Two positive clones of *Spli*Lec fragment were selected and sequenced (to exclude PCR errors certainly) using their specific forward and reverse primers (Table S1). Sequencing and sequence analyses were performed as described early in this section.

### Full-length cDNA isolation of immunolectin gene

Specific primers (sense and antisense) were designed based on the sequence of *Spli*Lec containing 3′ end. The 5′ end fragment was amplified using SMART RACE cDNA Amplification kit (Clontech) following the procedure outlined in the supplied user manual. The amplified 5′ end fragment was purified, cloned into *PCR-TOPO* vector, and sequenced as described early in this section. The sequences of 3′ and 5′ end fragments were aligned and the predicted full-length cDNA was obtained. Thus a pair of primers, LecFLF and LecFLR (Table S1), was designed for the amplification of full-length *Spli*Lec cDNA. PCR was carried out in a total volume of 25 µl reaction solution containing 2.5 µl PCR buffer, 1.5 mM MgCl_2_, 200 µM dNTPs, 1 U *Taq* DNA polymerase (AmpliTaq, Perkin-Elmer), 2.5 µl of 10 pmol of each primer and 2 µl cDNA using the following protocol: 94°C for 5 min (hot start) followed by 35 cycles of amplification (94°C for 1 min, 60°C for 1 min, 72°C for 1.5 min) and a final extention step at 72°C for 10 min. Full-length *Spli*Lec was visualized and eluted from the gel using GenClean Kit (Invitrogen Corporation, San Diego, CA, USA) following the manufacturer's instructions.

### Nucleotide sequence and sequence analyses

In addition to the above mentioned analyses, ExPasy Proteomics Server (http://expasy.org/tools) was used to calculate physico-chemical parameters of the translated peptide (ProtParam tool). Furthermore, post-translational modifications and topology predictions were investigated using SignalP, NetCGlyc, NetOGlyc, NetGlycate, YinOYang, OGPET, NetPhos, NetPhosK, Sulfinator, NetNES, SOSUI and TMpred tools. Moreover, Phylogenetic analyses of the nucleotide sequence and its deduced amino acids were done using Phylogeny.fr web service, One Click mode. Poorly aligned positions and divergent sequences were eliminated manually. Multiple alignment of available published lectin-related nucleotide sequences was done before phylogenetic analyses to approximate sequence lengths manually. 100% homologous sequences of the same species with different accession numbers were represented by only one sequence. The cloned DNA fragment was deposited in GenBank under the HQ603826 accession number.

### Expression of the *Spli*Lec and in-gel fluorescence detection of *O*-GlcNAc residues

*p*PROEXTM HTa Prokaryotic Expression System kit (Life technologies, USA) was used to clone the purified PCR product corresponding to mature *Spli*Lec peptide following the manufacturer's instructions. Charged *p*PROEXTM HTa vector was transformed into the competent *E. coli* strain DH_5_α provided with the kit. Gene expression was induced by IPTG as described by Goh *et al.*, [21]. Induced and non-induced as well as the purified protein samples were dissolved in sample buffer and analyzed on 12.5% SDS-PAGE. The expressed protein was affinity-purified on nickel-nitrilotriacetic acid Superflow resin (Qiagen, Germany) according to the manufacturer's protocol. In-gel fluorescence detection of the *O*-GlcNAcylated proteins (a chemoenzymatic labeling strategy) was carried out as described by Clark *et al.*, [22], using Click-iT™ *O*-GlcNAc Enzymatic Labeling System (Invitrogen) following the manufacturer's instructions.

### Quantitative protein determination

Total protein concentrations of control haemolymph, immunized haemolymph and purified *Spli*Lec were quantified spectrophotometrically using Bio-Rad protein assay kit (Bio-Rad, USA) following the manufacturer's protocol. Standard curve was constructed by using Bovine gamma globulin (BGG). The difference between control and treated samples was considered as accumulated lectin in the haemolymph (subtraction method). Haemolymph volumes were corrected for total protein concentration all over the agglutination and antibacterial experiments.

### Determination of the molecular mass of *SpliLec*

The molecular mass of *Spli*Lec was determined by gel filtration chromatography on Superdex-200 (2.6×60 cm, void volume: 318 cm3) (Bio Pilot Pharmacia) calibrated with carbonic anhydrase (29,000), ovalbumin (45,000), albumin (66,000), and phosphorylase-b (974,000) at room temperature and a flow rate of 2.6 ml/min. The column was then reequilibrated and eluted with a buffered insect saline (BIS pH: 7.9) consisting of 130 mM NaCl, 5 mM KCl, 1 mM CaCl_2_, and 0.01 mM Tris-HCl. The marker proteins were purchased from Sigma Chemical Co. The lectin sample (3 ml, concentrated to a volume of ca. 1 mg protein) was chromatographed on the column. Hemagglutination activity of the haemolymph was 1∶32 titer prior to application to the column. Column effluent was monitored at 280 nm and 2.5 ml fractions were collected. The amount of protein in the collected samples (making up peaks) was measured by a spectrophotometer at 280 nm.

Electrophoresis on SDS-PAGE was carried out by the method of Laemmli [23], using a 4.5% (w/v) acrylamide stacking gel and a 12.5% (w/v) acrylamide separating gel. The protein was dissolved in sample buffer with or without 2% (w/v) β-mercaptoethanol and then heated for 5 min at 95°C. Samples were electrophoresed and the gel was stained using Coomassie Brilliant Blue R_250_ (CBB). At very low concentration experiments, the gel was stained using PageSilver™ Silver Staining Kit (Fermentas, USA) following the manufacturer's instructions. The gel was calibrated using broad range molecular weight marker (Sigma Chemical Co. Switzerland).

### Hemagglutination, carbohydrate-binding specificity and effect of temperature assays

Erythrocytes from human blood groups A, B and O (RH^+^), tested sugars and glycosubstances were purchased from Sigma (Sigma Chemical Co. Switzerland). Formalinized rabbit, cow, sheep, guinea-pig, rat and mouse bloods were purchased from the Egyptian Organization for Biological Products and Vaccines (VACSERA), Cairo, Egypt. All erythrocytes were glutaraldehyde treated, trypsinized as described by Haq *et al.* [24], and suspended in Tris-buffered saline (TBS) (25 mM Tris-HCl, 137 mM NaCl and 3 mM KCl, pH 7.0) as a 10% suspension. For hemagglutination assay, erythrocytes were prepared as 2% suspension in TBS. Haemolymph and *Spli*Lec were serially diluted 2-fold with 25 µl of TBS containing 5 mM CaCl_2_ in 96-well V-shaped microtitration plates. Then 25 µl of 2% erythrocytes were added and mixed well. The plate was incubated for 1 h at 37°C. Agglutinated erythrocytes formed a diffuse mat, whereas unagglutinated erythrocytes formed a clear dot at the bottom of the well.

To test carbohydrate specificity for the immunized haemolymph and purified *Spli*Lec, the hemagglutination assay was conducted by mixing haemolymph or *Spli*Lec (1.0 µg/ml in TBS containing 5 mM CaCl_2_) with serial dilutions of various carbohydrates at room temperature for 30 min. Cow erythrocytes (2%) were then added, and the plate was incubated at 37°C for 1 h before scoring for agglutination [25].

The effect of temperature on the immunized haemolymph and the purified *Spli*Lec activity was also investigated, using 25 µl aliquots in TBS. Samples were kept at 4°C or heated, in a water bath for 1 h at 10, 20, 30, 40, 50, 60, 70, 80, 90 and 100°C. The sample was chilled on ice immediately after heat treatment. The agglutinating activitiy of lectin was assessed at room temperature against cow RBCs. The experiments were conducted using four replicates at three different times.

### Agglutination of bacteria and yeast by *Spli*Lec

Standard strains gram (−) *E. coli*, gram (+) *S. aureus* and the yeast, *S. cerevisiae* (Molecular Probes) live cells were resuspended in TBS pH 7.4 at a concentration of 1.1×10^6^ cells/ml (suspension adjusted to 1 Macfarland turbidity standard) and agglutinating activities of the purified *Spli*Lec, control and immunized haemolymph were assessed as described early.

### Antibacterial assay

*In vitro* antibacterial studies of the immunized haemolymph and purified mature peptide samples were carried out by the agar disk diffusion method with minor modifications [26], [27]. Five milliliters of 0.6% melted LB agar (52°C) were mixed with 100 µl of viable bacterial strain suspension (1.6×10^9^ cells/ml), and poured into a 9 cm plastic dish. Five microliters of each haemolymph and purified *Spli*Lec protein samples were applied to a 6 mm diameter paper disk and incubated at 37°C. Total protein concentration was quantified spectrophotometrically in both the control and the bacterial-challenged samples using Bio-Rad protein assay kit (Bio-Rad, USA) following the manufacturer's protocol. The difference between the control and the treated samples was considered accumulated *Spli*Lec in the immunized haemolymph (subtraction method). Standard curve was constructed by using BGG. Haemolymph volumes were corrected for total protein concentration (1 µg/µl) all over the experiment. The working solution of the purified *Spli*Lec was quantified to be 1 µg/µl all over the experiment. Penicillin (10 mg/disc; obtained from Sigma) and normal saline solution were used as positive and negative controls, respectively. *E. coli*, *P. vulgaris*, *K. pneumoniae*, *S. aureus* and *S. sanguinis* were used for testing the antibacterial activity. Inhibition zone diameters of five replicates were measured after 24 and 48 h. The degree of growth inhibition was quantified after 16 h by comparison with the growth inhibition resulting from the positive control.

## Results

### Differential display using primers corresponding to well known lectins

Differential display technique was used to characterize the genetic variation (at RNA level) between bacterial-challenged and control cotton leafworm, *S. littoralis*. Fig. (S1) shows the results of differentially displayed cDNAs of bacterial-challenged and control insects using 8 primers corresponding to previously characterized lectins (Table S1). Haemolymph samples were differentially displayed at 24, 48 and/or 72 h p.i. with *S. aureus*, *S. sanguinis*, *E. coli*, *P. vulgaris* and *K. pneumoniae* bacterial strains. It was observed that *S. aureus*-challenged insects died 24 h p.i., *E. coli*-challenged insects died 48 h p.i. and *S. sanguinis*-challenged insects died 72 h p.i. All insects died before sampling in the case of *P. vulgaris* and *K. pneumoniae*. Differential display results revealed that the average number of bands per sample was 4.3 bands for each amplification reaction. The total number of bands (transcripts) resolved in 1.5% agarose gel for both control and challenged insects was 124 (molecular size ranged from >1300 to ∼80 bp). Forty seven polymorphic bands (37.9%) were differentially displayed with 6 of the used primers. Five reproducible, infection-induced bands were cloned and sequenced using M_13_ universal primer. Analyses of the results revealed that a fragment of 640 bp was amplified within the open reading frame (*orf*) of a lectin gene. This fragment contained the complete 3′ end with a poly(A) tail, but it was not complete at the 5′ end (lacking starting codon, AUG at its 5′ end).

### RT-PCR amplification and cloning of the lectin gene

**Figure 1:**
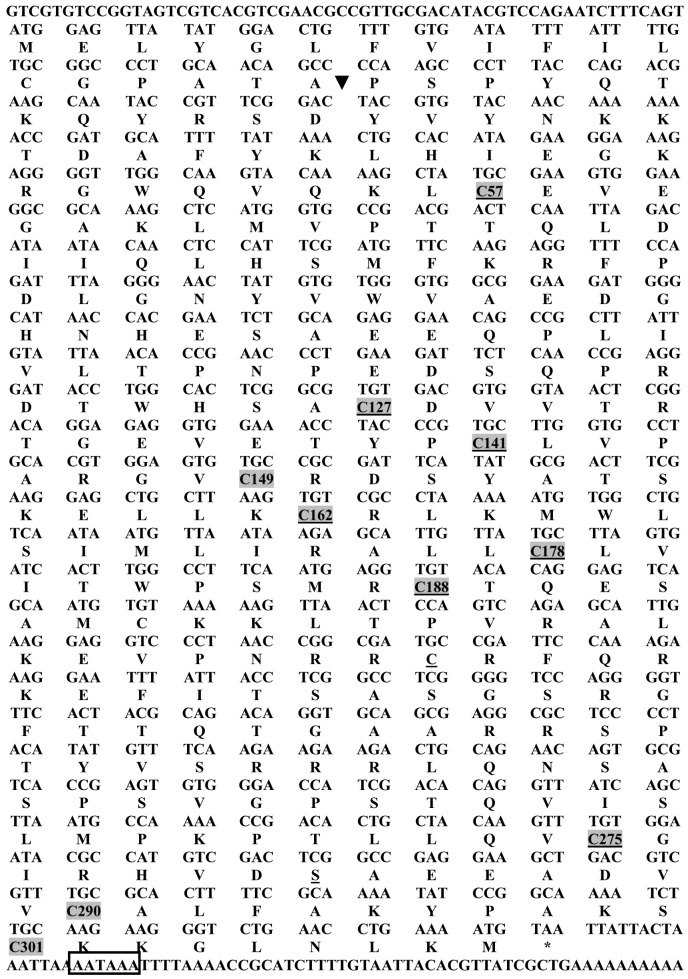
Nucleotide and corresponding deduced amino acid sequence of *S. littoralis* immunolectin gene (*Spli*Lec). Cleavage site between the signal and mature peptides are indicated by an arrow. Positions of cysteine residues are shaded. Asterisk indicates the stop codon. Boxed sequence represents the putative polyadenylation signal.

To obtain the full-length sequence, the 5′ end of the cDNA was amplified using RACE PCR method, purified, cloned and sequenced. The full-length sequence of *Spli*Lec cDNA was amplified using LecFLF and LecFLR. RT-PCR was optimized for the primer set and successfully amplified ≈1150 bp fragment (Fig. S2). The positive PCR product was visualized, eluted and cloned into *PCR-TOPO* vector (Fig. S2). Using PCR screening method, the clone *PCR-TOPOSpli*Lec was tested as positive (Fig. S2). Two positive clones of *Spli*Lec fragment were selected and sequenced (to exclude PCR errors certainly) using LecFLF and LecFLR primers (Fig. 1).

### Nucleotide sequence and sequence analyses

Nucleotide sequences of the *Spli*Lec and its deduced amino acid sequence is shown in Fig. (1). A single *orf* encoding a 309-residues polypeptide was detected in the *Spli*Lec sequence. One stop codon was found at the 3′ end. The flanking region of the initiation codon ATG is AGTATGGAG, and the length of 5′ untranslated region (UTR) was 60 bp before the start codon ATG. The length of 3′ UTR was 60 bp before the poly(A) track. The putative polyadenylation sequence AATAAA was located 15 bp downstream from the stop codon (Fig. 1). The identified *Spli*Lec *orf* includes a signal peptide (54 bp), and a mature peptide (873 bp). The deduced *Spli*Lec polypeptide contains 50 strongly basic, 28 strongly acidic, 127 hydrophobic and 104 polar uncharged amino acids. The calculated molecular masses of the putative *Spli*Lec and its mature peptide are 34.85 and 32.91 KDa, respectively, and the theoretical isoelectric points (PIs) were 9.27 and 9.38, respectively. The net charges at pH 7.0 were 15.9 and 16.9 for the *Spli*Lec and its mature peptide, respectively. Both the full length and the mature *Spli*Lec peptides were classified as unstable (Instability Index (II): 55.81 and 56.95, respectively). Ratios of the hydrophilic residues were calculated as 37 and 38% for the full length and its mature peptides, respectively.

**Figure 2:**
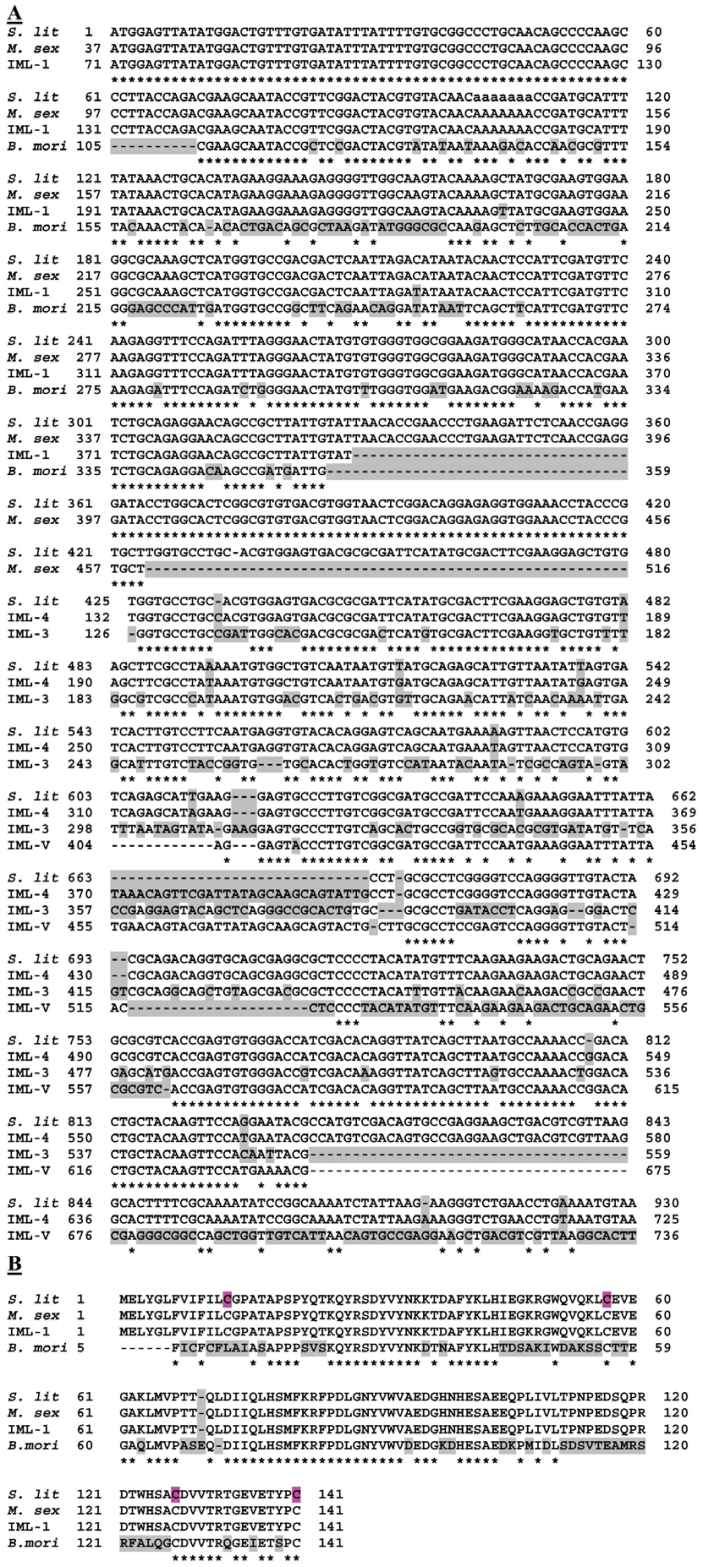
Comparison of the lectin nucleotide sequence of *S. littoralis* (Acc# HQ603826) with *M. sexta* immunolectin-A precursor, IML-1, IML-2, IML-3, IML-4, IML-V and *B. mori* C-type lectin (Acc# AF053131, GU454799, AF242202, AY768811, AY768812, AM293329 and AY297159, respectively). *S. lit*: *S. littoralis* lectin, *M. sex*: *M. sexta* immunolectin-A, IML-1: *M. sexta* immunolectin-1, IML-2: *M. sexta* immunolectin-2, IML-3: *M. sexta* immunolectin-3, IML-4: *M. sexta* immunolectin-4, IML-V: *M. sexta* immunolectin-V and *B. mori*: *B. mori* C-type lectin. Gaps and different nucleotides are shaded.

Nucleotide sequence and its deduced amino acid sequence of the *Spli*Lec were blasted with all available sequences in GenBank database. Alignment results revealed that the *Spli*Lec sequence (Acc# HQ603826) has a significant alignment with 9 and 14 published lepidopteran DNA and peptide sequences, respectively. Although the percentage identity ranged from 100% to 69% with IML-A precursor (Acc# AF053131) and IML-3 (Acc# AY768811) of *Manduca sexta*, it did not necessarily mean full consistence, especially when the percentage coverage of the gene was regarded. Some insect lectins covered the forward region of the *Spli*Lec sequence and others covered the backward segment (e.g. *M. sexta* and *Bombyx mori* immunolectins) (Fig. 2 A and B).

**Figure 3:**
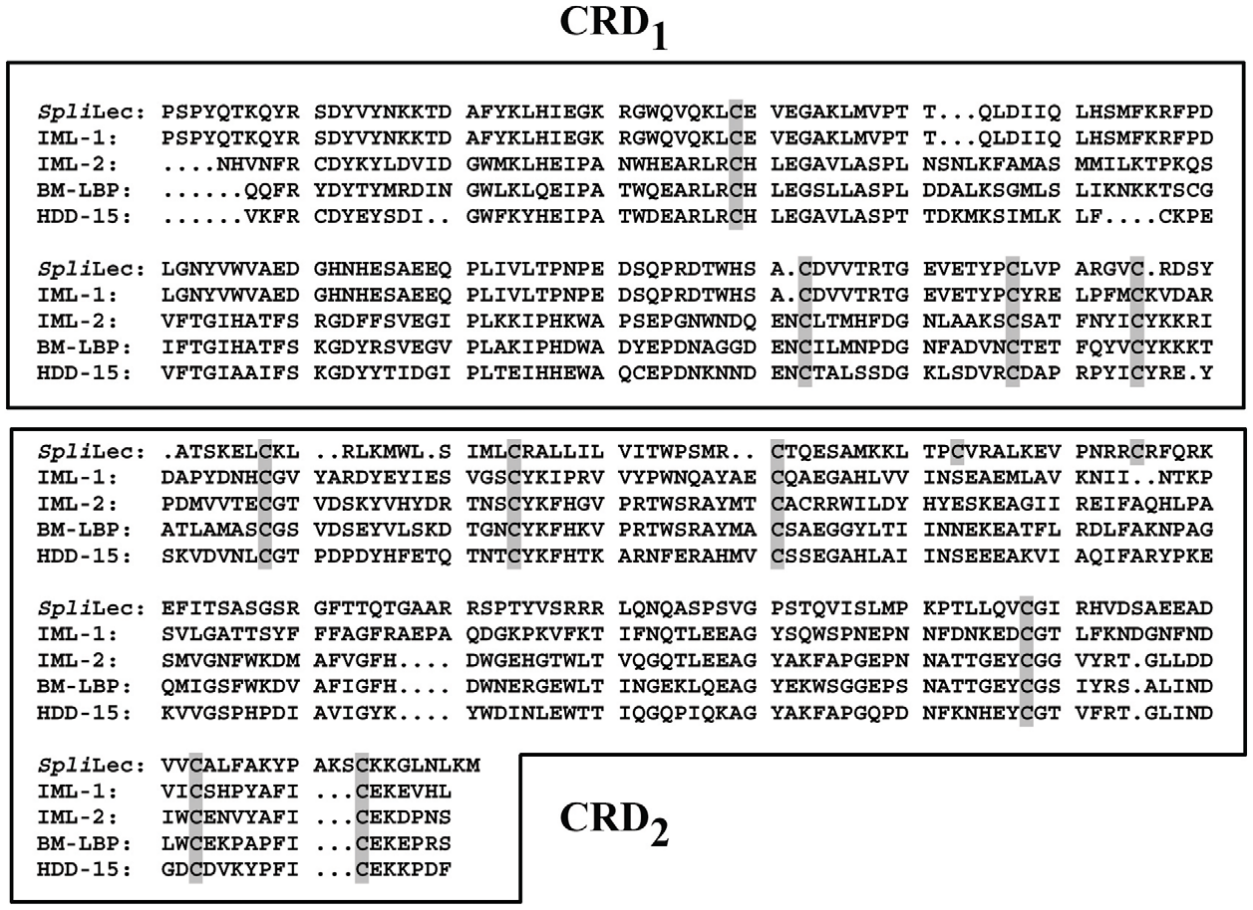
Alignment five mature polypeptide sequences of insect C-type lectins consisting of two CRDs. *Spli*Lec: *S. littoralis* lectin, *IML-1*: *M. sexta* immulectin-1, *IML-2*: *M. sexta* immulectin-2, *Bm-LBP*: *B. mori* LPS-binding protein, *Hdd15*: putative lectin from the fall webworm, *H. cunea*. The conserved four cysteine residues (2 disulphide bonds) in the short form CRD_1_ and the six cysteines (3 disulphide bonds) in the long form CRD_2_ are shaded.

Analysis of the amino acid sequence deduced from the cDNA indicated that *Spli*Lec is a member of the C-type lectin superfamily. It contains two C-type CRDs, an amino-terminal domain, CRD_1_ (residues 1–149), and a carboxyl-terminal domain, CRD_2_ (residues 160–301). Fig. (3) shows an alignment of the five insect C-type lectins with tandem CRD structure. *Spli*Lec shows 56%, 31% and 50% identities to *M. sexta* IML-1, IML-2 and IML-3 (with 31, 27 and 30 gaps), respectively. *Spli*Lec also shows 31% identity (2 gaps) to *B. mori* LPS-binding protein (BmLBP) and 29% identity (29 gaps) to Hdd15.

Primary, secondary structure analyses, post-translational modifications and topology predictions revealed that amino acid sequence of the putative *Spli*Lec peptide had one signal peptide cleavage site (between positions 18 and 19), one tyrosine-glycosylated and two tyrosine-sulfated sites at positions 111, 31 and 33, respectively. Fifteen *O*-GlcNAcylated residues (8 Ser and 7 Thr) and six potentially glycated lysines were predicted. Twenty one phosphorylation sites (Ser: 11, Thr: 6 and Tyr: 4) and 44 (24 S, 2 Y and 18 T) kinase specific phosphorylation sites (highest score: 0.82 PKC at position 185) were also predicted. In addition, two transmembrane helices (one primary: 166–182 with outside to inside orientation and one secondary: 3–22 with inside to outside orientation) were predicted.

### In-gel fluorescence detection of the *O*-GlcNAcylation of the *Spli*Lec

**Figure 4:**
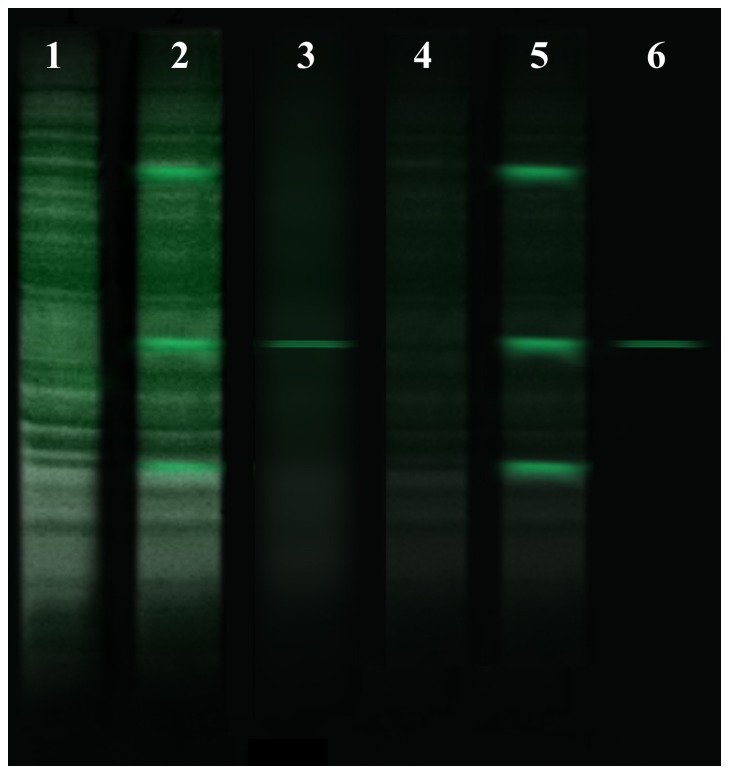
Direct in-gel fluorescence detection of *O*-GlcNAc modified proteins of the expressed and the purified *Spli*Lec protein after induction with IPTG. Lanes 1–3: control, induced and purified *Spli*Lec. Lanes 4–6: Western blotting using Click-iT™ kit.

Because the *O*-GlcNAc-modified proteins are some hard to predict, we confirm our predictions by some experimental evidences. The in-gel fluorescence results emphasized the identification of three unique *O*-GlcNAc-modified proteins in the case of bacterial-challenged haemolymph (*Spli*Lec and two additional proteins). Also, the purified *Spli*Lec was confirmed as *O*-GlcNAc-modified protein (Fig. 4, Lane 3). In-gel electrophoresis results were sustained by blotting results (Fig. 4, Lanes: 4–6).

### Phylogenetic analyses of lectin sequence

**Figure 5:**
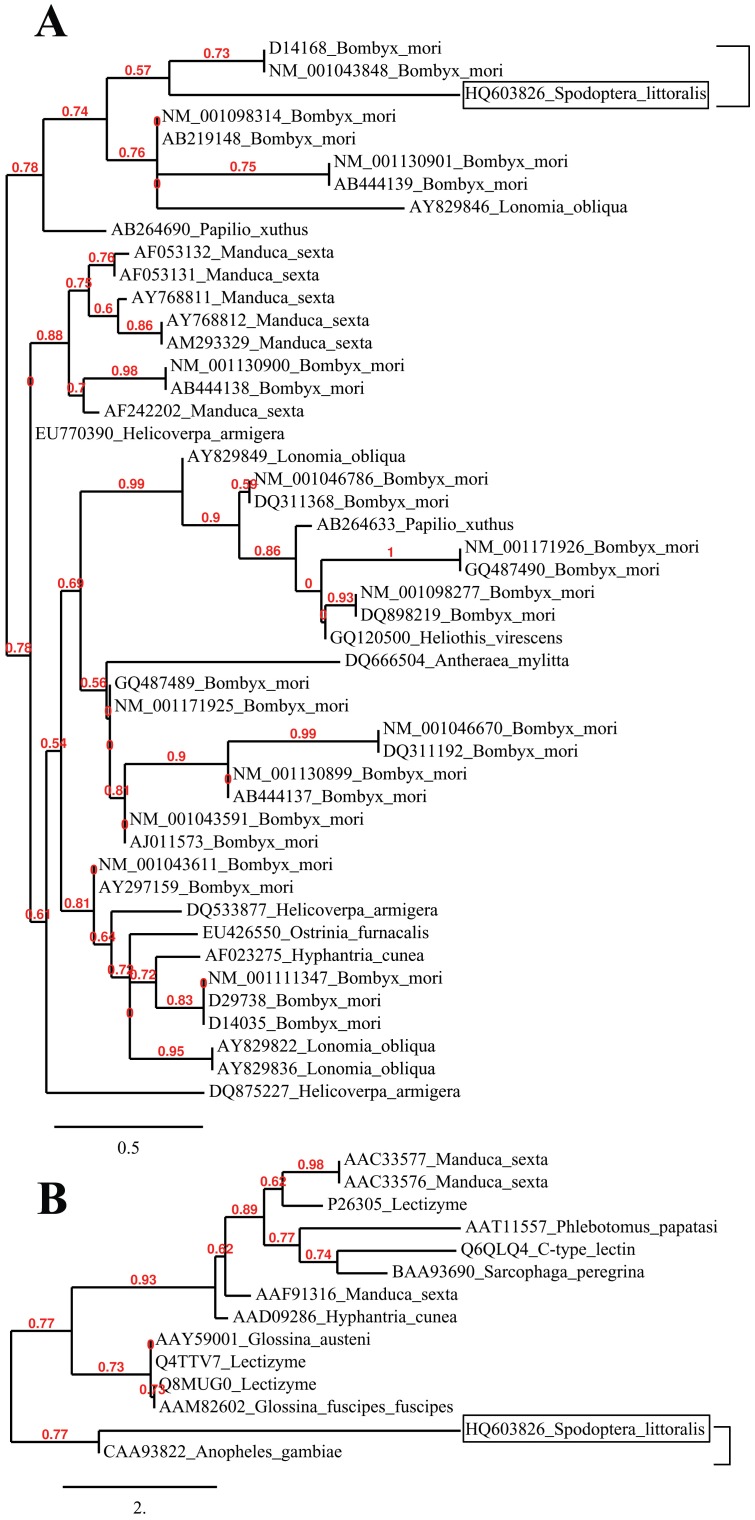
Phylogenetic analysis of *Spli*Lec nucleotide and deduced amino acid sequences compared to 46 and 13 sequences registered in NCBI. Phylogenetic trees were generated from 47 and 14 lectin-related sequences by neighbor-joining distance analysis using Phylogeny.fr web service, One Click mode. Full sequence names and accession numbers are included in the tree.

Phylogenetic analyses of the *Spli*Lec have been performed with the 47 nucleotide seuquence (including 10 insect genera from the order Lepidoptera.) and 14 polypeptides (including 8 insect species: 3 lepidopterans and 5 dipterans). The results of these analyses are shown in Figs. (5 A and B). Phylogenetic trees were generated by neighbor-joining distance analyses with maximum sequence difference 1.0. The nucleotide topology shows two distinct lineages including 9 (6 phylogenetic groups) and 38 (24 phylogenetic groups) lectin-related sequences, respectively. *Spli*Lec seuquence (Acc# HQ603826) was clustered in a monophyletic sister clade with 2 *B. mori* lectins (Acc# NM_001043848 and D14168) (Fig. 5A). The polypeptide topology shows two distinct lineages including 12 (7 phylogenetic groups) and 2 (1 phylogenetic group) lectin peptides, respectively. However in this case, *Spli*Lec polypeptide was clustered with *Anopheles gambiae* lectin (Acc# CAA93822) in the same lineage (Fig. 5B).

### Quantitative protein analysis

Quantitative protein analysis of the control and bacterial-challenged *S. littoralis* haemolymph was determined at 1, 6, 12, 24, 48 and 72 h p.i. (Table 1). Statistical analysis of data revealed that the increase of total protein content in case of bacterial-challenged insects was significant at all tested times. *Df*, *F* and *P* values were illustrated in Table (1). The expected antibacterial peptide concentration in the haemolymph of bacterial-challenged insects was increasing smoothly with the time and an abrupt peak was observed at 48 h p.i. In addition, the total protein concentration of IPTG-induced, non-induced transformed *E. coli* and Ni-affinity purified *Spli*Lec mature peptide was determined at 1, 2 and 3 h p.i. (Table 1). Protein concentration increased with the time course reaching maximum at 3 h p.i. Statistical analysis of data revealed that the difference of protein content (expressed protein) in case of IPTG-induced and non-induced cells was significant at the tested times. *F* and *P* values were illustrated in Table (1). The quantity of protein lost by purification (loss due to purification = induced – non-induced – purified) was 60, 114.4 and 80.6 µg at 1, 2 and 3 h p.i., respectively. This loss was statistically significant (*P* = 0.00) at all the tested cases.

**Table 1:** Quantitative protein analysis of the crude haemolymph of *S. littoralis* and expressed antibacterial peptide after induction of the recombinant *E. coli* by IPTG.

Protein concentration at different hours post-infection or post-induction (µg/ml) Mean ± S.E.								
	1 h	2 h	3 h	6 h	12 h	24 h	48 h	72 h
Cont-H	596.6±15.2	-	-	669.8±20.2	638.4±27.3	663.0±13.2	653.4±10.0	644.8±5.7
Inf-H	683.8±14.0	-	-	875.6±15.7	921.0±8.7	916.2±15.9	1965.6±29.3	1476.6±29.8
Expec. Lec.	87.2	-	-	205.8	282.6	253.2	1312.2	831.8
Induced	922.2±21.2	1095.4±48.9	1413.2±38.9	-	-	-	-	-
NonInd	711.4±14.9	720.2±8.8	918.8±26.2	-	-	-	-	
Expressed P.	210.8±3.8	375.2±24.4	494.4±36.2					
Purified	146.4±8.7	255.4±26.7	413.8±8.1	-	-	-	-	-
Loss	60.0±3.3	114.4±26.8	80.6±18.4					
*df*, *F*, *P*	5, 581.46, 0.0	-, 205.84, 0.0	-, 522.52, 0.0	4, -, 0.003	4, -, 0.0	4, -, 0.0	4, -, 0.0	4, -, 0.0

**Cont-H : Untreated crude haemolymph.**

**Inf-H : Treated haemolymph.**

**Expec. Lec. : Expected lectin concentration.**

**Induced: IPTG induced** ***E. coli*** **culture.**

**NonInd : Non-induced** ***E. coli*** **culture.**

**Expressed P.: (Induced – Non-induced).**

**Purified : Purified expressed peptide using Ni-affinity column.**

**Loss: Loss due to purification.**

### Molecular mass determination of the *Spli*Lec

**Figure 6:**
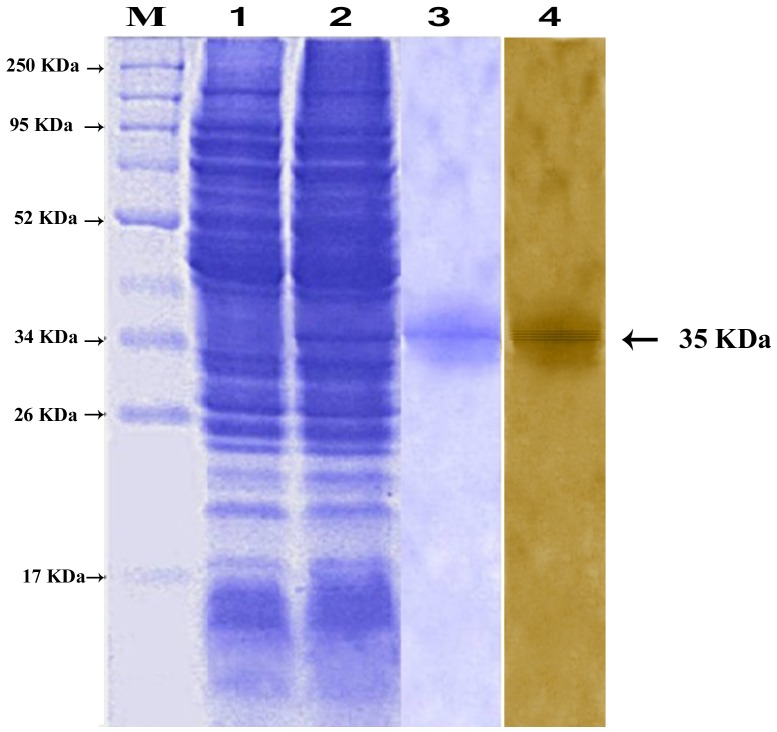
12.5% SDS-PAGE showing the expressed and the purified *Spli*Lec protein after induction with IPTG. Lanes 1–4: non-induced, IPTG-induced *E. coli*, purified *Spli*Lec (CBB-stained), purified *Spli*Lec (silver-stained). Lane M: broad-range protein marker. Molecular weight of the marker is indicated as KDa.

The affinity purified *Spli*Lec exhibited an apparent molecular mass of 140 kDa as determined by gel filtration chromatography. In SDS-PAGE at pH 8.3, *Spli*Lec gave a single band with a subunit molecular mass of 35 kDa under reducing and non-reducing conditions (Fig. 6). At very low concentrations (0.5 ng/5 µl sample), the single band separated as four sharp discrete bands using silver staining method (Fig. 6).

### Assay of hemagglutinating activity of *Spli*Lec

A hemagglutination assay was performed to test the ligand binding specificity of *Spli*Lec using various red cells in the presence of 1 mM CaCl_2_ is shown in Table (2). *Spli*Lec agglutinated cow erythrocytes most effectively; followed by human group A and B erythrocytes and it showed minimal activity with rabbit erythrocytes (Table 2). The crude haemolymphs of control and bacterial-immunized insects agglutinated cow erythrocytes, followed by human group B and A erythrocytes, and finally sheep erythrocytes (Table 2). The hemagglutinating activity of *Spli*Lec was inhibited by the addition of EDTA to the reaction.

**Table 2:** Hemagglutinating activity of the purified *Spli*Lec mature peptide, control and immunized haemolymphs of *S. littoralis* on erythrocytes.

Erythrocytes	Minimum agglutinating concentration (µg/ml)						
	Control haemolymph			Immunized haemolymph			Purified *Spli*Lec
	24 h	48 h	72 h	24 h	48 h	72 h	
**Human A+**	NA*	80	NA	80	10	10	2.5
**Human B+**	NA	80	80	40	5	5	2.5
**Human O+**	NA	NA	NA	NA	NA	NA	40
**Rabbit**	NA	NA	NA	NA	NA	NA	80
**Cow**	80	40	40	20	2.5	2.5	0.6
**Sheep**	NA	40	40	NA	20	40	20
**Guinea-pig**	NA	NA	NA	NA	NA	NA	40
**Rat**	NA	NA	NA	NA	NA	NA	40
**Mouse**	NA	NA	NA	NA	NA	NA	40

The assay was performed at concentrations: 80, 40, 20, 10, 5, 2.5, 1.25, 0.6, 0.3 and 0.15 µg/ml.

* **NA: No activity at 80 µg/ml.**

The inhibition of agglutination of cow erythrocytes was tested to identify carbohydrates that compete with erythrocytes in binding to *Spli*Lec. The activity of the *Spli*Lec was most effectively inhibited by galactose or oligosaccharides containing galactose (*N-*acetylgalactosamine, raffinose and lactose); followed by mannose, glucose and *N*-acetylglucosamine and xylose. Weaker inhibiting effect, or none at all, was detected when the other sugars were used (Table 3). Among polysaccharides tested, laminarin (β-1,3-glucan) inhibited the agglutinating activity of *Spli*Lec more effective than mannan (polymer of mannose). Similar results were obtained when the immunized haemolymph containing *Spli*Lec was examined (Table 3).

**Table 3:** Competing effects of sugars on agglutinating activity of the purified *Spli*Lec mature peptide and immunized haemolymph of *S. littoralis* against cow red blood cells.

Saccharides	Minimum inhibitory concentration (mM or µg/ml)			
	Immunized haemolymph			Purified *Spli*Lec
	24 h	48 h	72 h	
**D-Xylose**	100	100	NI	50
**D-Glucose**	50	50	100	30
**D-Mannose**	NI^a^	50	NI	10
**D-Galactose**	50	50	100	3
***N-*** **Acetylglucosamine**	100	100	NI	30
***N-*** **Acetylgalactosamine**	50	50	100	10
**Maltose**	NI	100	100	100
**Sucrose**	NI	NI	NI	100
**Raffinose**	50	30	100	10
**Lactose**	50	50	100	10
**Trehalose**	NI	NI	NI	NI
**Fructose**	NI	NI	NI	100
**Mannan**	NI^b^	NI	NI	100
**Laminarin (β-1,3-glucan)**	50	50	100	1

The assay was performed with sugars at concentrations: 1, 3, 10, 30, 50 and 100 mM and with glycosubstances at concentrations: 1000, 100, 50, 30, 20, 10, 5, 2.5 and 1 µg/ml.

**a: Not inhibited at 100 mM.**

**b: Not inhibited at 1 mg/ml.**

The hemagglutination activity was reduced to 50, 65, 69 and 75% when the immunized haemolymph was incubated with cow erythrocytes at 50, 70, 80 and 100°C, respectively, for 1 h. However, the exposure of the purified *Spli*Lec to the different temperatures had no effect on its agglutinating activity against cow erythrocytes (Table 4).

**Table 4:** Effect of temperature on agglutinating activity of the purified *Spli*Lec mature peptide and immunized haemolymph of *S. littoralis* against cow red blood cells.

Temprature	Minimum inhibitory concentration (µg/ml)			
	Immunized haemolymph			Purified *Spli*Lec
	24 h	48 h	72 h	
**10°C**	25	25	40	0.6
**20°C**	25	25	40	0.6
**30°C**	25	25	40	0.6
**40°C**	25	25	40	0.6
**50°C**	50	50	70	0.6
**60°C**	50	50	70	0.6
**70°C**	70	70	80	0.6
**80°C**	80	80	80	0.6
**90°C**	80	80	NA*	0.6
**100°C**	100	NA	NA	0.6

The assay was performed at concentrations: 80, 40, 20, 10, 5, 2.5, 1.25, 0.6, 0.3 and 0.15 µg/ml.

* **NA: No activity at 80 µg/ml.**

In order to test whether *Spli*Lec can bind to the surface of microorganisms, an agglutination assay was performed using *E. coli* gram (−) bacteria, *S. aureus* gram (+) bacteria and *S. cerevisiae* (yeast) (Table 5). The agglutinating activity of the control haemolymph was observed only in the case of *S. aureus* gram (+) bacteria. However the immunized haemolymph agglutinated the three tested microorganisms at 48 h p.i. A greater binding activity of the purified *Spli*Lec against the three tested microorganisms was observed even at very low concentrations (0.1 to 0.6 µg/ml) (Table 5). The agglutinating activity of the purified *Spli*Lec was inhibited by the addition of EDTA to the reaction.

**Table 5:** Agglutinating activity of the purified *Spli*Lec mature peptide, control and immunized haemolymphs of *S. littoralis* against bacteria at 24, 48 and 72 h post-infection.

Microbe	Minimum agglutinating concentration (µg/ml)						
	Control haemolymph			Immunized haemolymph			Purified *Spli*Lec
	24 h	48 h	72 h	24 h	48 h	72 h	
***E. coli*** ** (−)**	NA	NA	NA	NA	80	80	0.3
***S. aureus*** ** (+)**	NA	80	NA	80	40	80	0.1
***S. cerevisiae*** ** (Yeast)**	NA	NA	NA	NA	80	NA	0.6

The assay was performed at concentrations: 80, 40, 20, 10, 5, 2.5, 1.25, 0.6, 0.3 and 0.15 µg/ml.

**NA: No activity at 80 µg/ml.**

### Antibacterial assay

Table (6) shows a summary of the antimicrobial screening of the immunized haemolymph and the Ni-affinity purified mature *Spli*Lec peptide (Fig. 6) based on the microbial growth inhibition zone (in mm). Significant antibacterial activity of the immunized haemolymph and the purified *Spli*Lec was observed against the tested gram (+) bacteria (Table 6). Notably the antibacterial activity of the purified *Spli*Lec 48 h p.i. was more than 24 h p.i. for all the tested bacteria. As for the activity of the immunized haemolymph 24 and 48 h p.i., no difference was observed in the case of *P. vulgaris* and *K. pneumoniae*. The antibacterial activity of the immunized haemolymph and the purified *Spli*Lec was less than the positive control in the case of *P. vulgaris*. However, the activity was more than or comparable to the positive control in the case of the other tested bacteria, 48 h p.i. (Table 6).

**Table 6:** Antibacterial activity of the purified *Spli*Lec mature peptide and the immunized haemolymph of *S. littoralis* on gram (−) and gram (+) bacteria.

Microorganism (Gram stain)	Antibacterial activity			
	Immunized haemolymph		Purified *Spli*Lec	
	24 h p.i.	48 h p.i.	24 h p.i.	48 h p.i.
***Escherichia coli*** ** (−)**	**+**	**++**	**+**	**++**
***Proteus vulgaris*** ** (−)**	+	**+**	(+)	+
***Klebsiella pneumoniae*** ** (−)**	**+**	**+**	**+**	**++**
***Staphylococcus aureus*** ** (+)**	**++**	**+++**	**++**	**+++**
***Streptococcus sanguinis*** ** (+)**	**++**	**+++**	**+++**	**+++**

(+): Inhibition zone less than 1 mm surrounding the 6 mm paper disk.

+: Inhibition less than positive control.

++: Inhibition comparable to positive control.

+++: Inhibition more than 10 mg penicillin; inhibition zones of references = 13±1 mm diam.

## Discussion

In the present study, the common bands revealed by DD-PCR in both control and challenged samples may represent the house-keeping genes. Some bands were recorded in control insects and disappeared in challenged ones (genes were turned off). On the other hand, many bands were induced as a result of bacterial-challenge at different time intervals post-infection. DD-PCR technique is considered a powerful genetic screening tool for complicated dynamic tissue processes [28], to detect and compare altered gene expression in eukaryotic cells [29], to screen and to characterize differentially expressed mRNAs [30], because it allows for simultaneous amplification of multiple arbitrary transcripts. Many publications described the enhancement of the insect immune system and induction of lectins due to stress and/or infection (*e.g.* [15], [31]–[33]). Lectins were isolated from six insect orders: Lepidoptera (*e.g.* [3], [8], [15]–[19], [32], [34]), Diptera (*e.g.* [1], [24], [35]), Coleoptera (*e.g.* [36]), Hemiptera (*e.g.* [2], [37]), Orthoptera (*e.g.* [38]) and Dictyoptera (*e.g.* [39]). As the C-type lectins are important molecules in the innate immune systems, we isolated the full-length cDNA of *S. littoralis* lectin (*Spli*Lec) which shares typical features of the C-type insect lectins.

The full-length cDNA of the *Spli*Lec was 1150 bp, a size very similar to that of *M. sexta* IML-2 [14], and contained a 927 bp *orf* encoding 309 amino acids. The flanking region of the *Spli*Lec initiation codon ATG keeps the adenine nucleotide at position -3 which is a universal feature in all the eukaryote genes [40]. Like many insect lectins, the *Spli*Lec was predicted to have a 18-residue secretion signal peptide and a 291-residue mature protein. The deduced amino acid sequence of *M. sexta* IML-2 was reported to contain a 19-residue secretion signal peptide and a 308-residue mature protein [14]. *Sarcophaga* C-type lectin was predicted to have a 23-residue secretion signal peptide and a 150-residue mature protein [35]. Signal secretion peptides were reported to be 21-, 21- and 26-residue in the cases of *Drosophila* DL1, DL2 and DL3 C-type lectins, respectively [41]. It is notable that the *Spli*Lec gene also shares homology to many C-type insect lectins and it consists of two CRDs: the amino-terminal CRD_1_ is short form, with two intramolecular disulfide bonds (Cys^57^–Cys^127^ and Cys^141^–Cys^149^) and the carboxyl-terminal CRD_2_ is long form, with three intramolecular disulfide bonds (one additional disulphide bond near the amino terminus: Cys^162^–Cys^178^). This feature of the *Spli*Lec is similar to the two immulectins of *M. sexta* (IML-1 and IML-2) [42], LPS-binding proteins of the silkworm, *B. mori* [17] and the putative lectin of the fall webworm, *H. cunea* [18].

Reconstruction of the phylogenetic trees of the *Spli*Lec nucleotide sequence and its deduced polypeptide resulted in two different topologies. Both of the two trees clustered *Spli*Lec sequence in two different groups (clustered with *Bombyx* in the case of nucleotide-based tree and with *Anopheles* in the case of amino acid-based tree) indicating the possibility of evolutionary trend between these lectins which might descend from a common ancestor. Grouping of some lepidopteran and dipteran lectins (*e.g. M. sexta* with *Sarcophaga* and *S. littoralis* with *Anopheles*) in one sister clade indicated that they may be homologous or share some similarity. In addition, lepidopteran lectin-like sequences were diverged in many sister clades as amino acids due to the difference in codon usage in different species.

The predicted post-translational modifications of the *Spli*Lec protein suggested an important role of the *Spli*Lec protein in modulating a broad range of biological processes in the cell. The predicted and experimentally confirmed O-GlcNAcylation suggested a possible function of the *Spli*Lec protein in macromolecular complex assembly and intracellular transport. Glycosylation and glycation serve for the correct folding and stability of the protein (unglycosylated proteins degrade quickly). Glycosylation of proteins play a role in cell-cell adhesion (a mechanism employed by cells of the immune system), as well [43]. Reversible phosphorylation of proteins (using kinases and phosphatases) is considered an important regulatory mechanism in protein-protein interaction via recognition domains, (i.e. many proteins and receptors are switched “on” or “off” by phosphorylation and dephosphorylation). It also results in a conformational changes in the structure in many peptides, causing them to become activated, deactivated or degraded [44]. In addition, many transmembrane proteins (TPs) function as gateways or “loading docks” to deny or permit the transport of specific substances across the biological membranes (to get into or out of the cell by folding up or bending through the membrane). Some of these functions may introduce a model that explains the antimicrobial and agglutinating activity of the *Spli*Lec.

The molecular weight of invertebrate lectins varies from 26 to 1500 KDa (*e.g.* [8], [10]–[14], [42], [45]). This variation may be due to the difference of species, method of purification and analysis of lectins. Lepidopteran lectin-like molecules include both single and several subunit lectins. However, some lectins lack subunits [45]. Based on SDS-PAGE and gel filtration results, the *Spli*Lec protein was shown to be a tetrameric lectin with a subunit molecular mass of 35 kDa. This result is consistent with the calculated molecular mass of the *Spli*Lec (34.9 KDa). Further confirmation was achieved when the highly diluted samples (0.5 ng/5 µl) were electrophoresed and the gel was silver-stained. The four monomers (subunits) of the tetrameric lectin dissociated and obviously stained as four sharp discrete bands. The *Periplaneta americana* purified lectin was detected to be the largest molecular weight insect lectin (1500 KDa with a subunit molecular weight of 30 KDa on SDS-PAGE), whereas the lowest molecular weight lectin was that of *A. segetum* (69 KDa with no subunits) [45]. Gül and Ayvali [45] also reported the molecular weights and subunits of many previously determined lepidopteran lectins: *Antheraea pernyi* lectin was 380 KDa with a subunit molecular weight of 38 KDa; *Hyalophora cecropia* lectin was 160 KDa with 41 KDa α-subunit and a 38 KDa β-subunit; and *Spodoptera exigua* lectin was 100–700 KDa with 32.2–34.4 KDa two subunits. The partially purified haemolymph lectin from the fifth instar larvae of *Bombyx mori* was 260 KDa or 350 KDa with no subunits [45]. This difference between the molecular weights of the lectin of the same insect species may be due to the difference in preparation procedures. Molecular weights and subunits of dipteran lectins were determined too: the *Sarcophaga peregrina* lectin was shown to be 190 KDa (four α subunits of 32 KDa and two β subunits of 30 KDa) [45]; *Calliphora vomitoria* lectin was 130 KDa with 32 KDa subunits [45] and *Culex quinquefasciatus* lectin was 34.5 KDa using SDS-PAGE [46]. Finally the migratory grasshopper, *Melanoplus sanguinipes*, lectin was reported to be of 600–700 KDa on SDS-PAGE and gel filtration [45].

Although the C-type lectin family includes members that bind their ligands in a calcium-dependent manner, many other C-type lectins show the same activity in a calcium-independent manner. The present study clarified that calcium was essential to the hemagglutinating and microbial aggregating activities of the *Spli*Lec peptide confirming that it is a calcium-dependant C-type lectin. In contrast, the IML-2 of *M. sexta* did not require calcium for its binding activity [14]. The agglutination of cow erythrocytes by the *Spli*Lec was not affected by heating to 100°C, confirming its thermal stability. This result confirmed the results obtained by Santos *et al.*, [47] who isolated a lectin which was thermostable at 100°C during 7 h. Thermostable lectins were also reported in the coleopteran *Allomyrina dichotoma* [36], the orthopteran *L. migratoria* [48] and the culicid *C. quinquefasciatus* [46]. However, thermal instability is a characteristic of lectins of some other insects, e.g. the orthopteran *T. commodus* [49], the dipteran *Glossina fuscipes* [50]. In addition, the agglutination of cow erythrocytes by the *Spli*Lec was inhibited most efficiently by the monosaccharide galactose or oligosaccharides containing galactose (*N-*acetylgalactosamine, raffinose and lactose); followed by mannose, glucose and N-acetylglucosamine and xylose. Xylose is a pentose, whose 2-, 3-, and 4-hydroxyl groups have the same configurations as those in glucose. Mannose differs from glucose only at the configuration of 2-OH, whereas galactose differs from glucose at the 4-OH. These results suggest that the 2-, 3-, and 4-hydroxyl groups of monosaccharides may participate in the binding to CRDs of the *Spli*Lec. These binding properties are consistent with the predicted binding sites in CRD_1_ (Glu^94^, Gly^96^ and Gln^70^, Asp^72^) and CRD_2_ (Glu^206^ and Asn^209^) of the *Spli*Lec amino acid sequence. Glu-Gly residues can interact with the sugar by hydrogen bonding to the equatorial 3-OH and 4-OH groups of mannose, glucose or other sugars with similar adjacent equatorial hydroxyls [51]. In addition, the Gln-Asp residues can bind galactose (or similar sugars with an axial 3-OH and equatorial 4-OH) [51]. In CRD_2_ of the *Spli*Lec, Glu-Asn residues would be predicted to bind mannose or glucose. Future studies on the carbohydrate binding activity of the *Spli*Lec are needed to sustain the surprising results obtained in this section (using techniques which do not rely on the inhibition of agglutination).

The purified *Spli*Lec agglutinated both gram (+), gram (−) bacteria and yeast, as well. Similar results were observed with IML-1 of *M. sexta* [3]. Weaker activity was observed with the IML-2 of *M. sexta* [14]. The four critical residues for ligand binding specificity in CRD_1_ of *Spli*Lec are Glu, Gly, Gln and Asp, with predicted specificity for galactose, glucose, mannose or other similar sugars as discussed above. However, these critical residues differ in CRD_2_ of the *Spli*Lec to be Glu-Asn residues would be predicted to bind mannose or glucose. The IML-1 and IML-2 of *M. sexta* have different critical residues for ligand binding specificities in their CRD_1_ (Gln and Arg in IML-1 and Glu and Gly in IML-2) and CRD_2_ (Glu and Asn). Perhaps these differences may lead to a broader or narrower ligand binding specificities. The polysaccharide laminarin (β-1,3-glucan) was the most efficient inhibitor of erythrocyte agglutination by the *Spli*Lec. Also, the purified *Spli*Lec agglutinated both gram (+), gram (−) bacteria and yeast. These results point toward laminarin (a component of the cell wall of *S. cerevisiae*) as a ligand of the *Spli*Lec and a function in recognition of bacterial and yeast membranes. Yu *et al.* [42] reported that the *M. sexta* immulectin could bind to bacterial lipopolysaccharide (LPS), lipoteichoic acid (LTA) and fungal β-1,3-glucan. IML-2 of *M. sexta*, *B. mori* LPS-binding protein and the individual recombinant CRDs of *H. cunea* lectin have been shown to bind to bacterial LPS [3], [14]. Yu *et al.* [52] further recorded the binding specificity of IML-2 of *M. sexta* to bacterial lipid A, several smooth and rough mutants of LPS and peptidoglycan, as well as to fungal mannan and β -1, 3-glucans (laminarin and curdlan). β-glucans (e.g. laminarin) are known as “biological response modifiers” because of their ability to activate the immune system [53]. Consequently, the lectins are probably acting as bridging molecules, by binding to the external polysaccharides of the bacterial and yeast membranes and then to receptors on the surface of the plasmatocytes [53]. Many insect immune peptides are active against gram (+) bacteria. However, the purified mature *Spli*Lec and the immunized haemolymph displayed a remarkable antibacterial activity against both gram (+) and gram (−) bacteria. Most C-type lectins are able to bind microorganisms themselves through recognizing carbohydrate, so as to directly be involved in innate defense mechanisms as a part of the acute-phase response to infection [54]. In addition to the traditional antimicrobial proteins (AMPs), such as defensin [31], several C-type lectins have been reported to have antibacterial activity. In invertebrates, the C-type lectin purified from the tunicate *Polyandrocarpa misakiensis* displayed a strong antibacterial activity even at the concentration of 1 µg/ml [55]. The recombinant protein of the scallop CFLec-1 displayed a remarkable inhibiting effect on gram (+) bacteria *Micrococcus lutens* and relatively weak lytic activity against gram (−) bacteria *E. coli* JM109 [56]. Riera *et al.* [57] reported a strong bacteriostatic activity against *E. coli*, *P. morganii* and *Enterococcus faecalis*. In short, these findings shed a new light on the lectin-mediated immune system. Combination of our findings with that reported by Seufi *et al.* [31] suggested that the *Spli*Lec and *Spli*Def peptides with other possible AMPs may constitute the defense network of *S. littoralis* against almost all possible invading microorganisms.

Conclusively, our current results provide a new insect lectin gene (*Spli*Lec) with a two tandem CRDs. The *Spli*Lec plays an important immune role in *S. littoralis* by cooperating with other AMPs to clear bacterial invaders. These findings would be helpful in future studies on lectins concerning ELISA, PCR and other related molecular and immunological techniques. In future, we are going to complete studies on the carbohydrate-binding activity using the high technology of glycan array and on the determination of the three-dimensional structure of the *Spli*Lec to provide a direct evidence for carbohydrate-binding mechanisms by its CRDs.

## Supporting Information

Figure S1 **Representative 1.5% agarose gels of the DD-PCR patterns generated from control and** ***S. aureus*** **,** ***E. coli*** **and** ***S. sanguinis*** **-challenged haemolymph samples using 8 primers corresponding to well known lectin genes.** Lane M: DNA marker 100 bp Ladder, lanes 1, 4, 8 and 10: controls of different treatments, lanes 9 and 11: 24 h post-infection with *S. aureus*, lanes 2, 3 and 5, 6: 24 and 48 h post-infection with *E. coli* and lane 7: 72 h post-infection with *S. sanguinis*. Arrows refer to differentially displayed sequenced bands.
(TIF)

Figure S2 **Agarose gel electrophoresis showing positive PCR representing the full length** ***Spli*** **Lec (1150 bp), clone** ***PCR-Spli*** **Lec, clone** ***PCR-Spli*** **Lec after insert release with** ***EcoR*** **I, and PCR confirmation.** Lanes 1, 2, 3, 4 and 5 show *Spli*Lec PCR product (1150 bp), *E. coli* harbouring *PCR-Spli*Lec, *PCR-Spli*Lec after digestion with *EcoR*I, positive control (*Spli*Lec amplified from the cDNA), and negative control (PCR mix without cDNA). Lane M: DNA Marker. The size of the bands is shown in bp.
(TIF)

Table S1 Key table for the primers used in this study providing their names, origin and sequences.
(DOC)
