# Supplementary figures and images for: Characterization of Multisugar-Binding C-Type Lectin (SpliLec) from a Bacterial-Challenged Cotton Leafworm, Spodoptera littoralis
Source: PLoS One. 2012 Aug 20;7(8):e42795. doi: 10.1371/journal.pone.0042795 (PMC3423437; doi:10.1371/journal.pone.0042795)

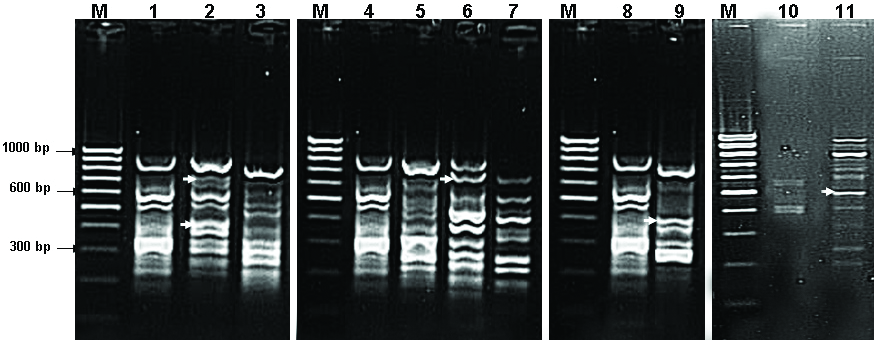

Supplement: Figure S1 — Representative 1.5% agarose gels of the DD-PCR patterns generated from control and S. aureus , E. coli and S. sanguinis -challenged haemolymph samples using 8 primers corresponding to well known lectin genes. Lane M: DNA marker 100 bp Ladder, lanes 1, 4, 8 and 10: controls of different treatments, lanes 9 and 11: 24 h post-infection with S. aureus, lanes 2, 3 and 5, 6: 24 and 48 h post-infection with E. coli and lane 7: 72 h post-infection with S. sanguinis. Arrows refer to differentially displayed sequenced bands. (TIF) [file pone.0042795.s001.tif]

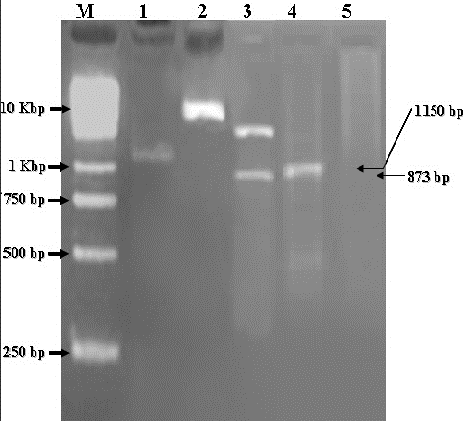

Supplement: Figure S2 — Agarose gel electrophoresis showing positive PCR representing the full length Spli Lec (1150 bp), clone PCR-Spli Lec, clone PCR-Spli Lec after insert release with EcoR I, and PCR confirmation. Lanes 1, 2, 3, 4 and 5 show SpliLec PCR product (1150 bp), E. coli harbouring PCR-SpliLec, PCR-SpliLec after digestion with EcoRI, positive control (SpliLec amplified from the cDNA), and negative control (PCR mix without cDNA). Lane M: DNA Marker. The size of the bands is shown in bp. (TIF) [file pone.0042795.s002.tif]
