# Supplementary material for: Characterization of Multisugar-Binding C-Type Lectin (SpliLec) from a Bacterial-Challenged Cotton Leafworm, Spodoptera littoralis
Source: PLoS One. 2012 Aug 20;7(8):e42795. doi: 10.1371/journal.pone.0042795 (PMC3423437; doi:10.1371/journal.pone.0042795)
Supplement: Table S1 — Key table for the primers used in this study providing their names, origin and sequences. (DOC) [file pone.0042795.s003.doc]

**Table S1: Key table for the primers used in this study providing their names, origin and sequences.**

| **Primer name** | **Origin** | **Sequence (5` -- 3`)** |
| --- | --- | --- |
| **LecF_1_** | **Lectin-based** | **AGTGGTAACAACGCAGAGTACGCGGGGG** |
| **LecR_1_** | **Lectin-based** | **ATATTTTAAAATCATCTCGTGTCCGGC** |
| **LecF_2_** | **Lectin-based** | **ATGGGATCCAAGCAACAGAG** |
| **LecR_2_** | **Lectin-based** | **ATCCTTCAAAGACACAATGTCG** |
| **Fwd_1_** | **Lectin-based** | **CTCACTGTGAATATGAAAGCGGCGA** |
| **Fwd_2_** | **Lectin-based** | **CTGACGTCCACACGTAAAATGATGTGT** |
| **Fwd_3_** | **Lectin-based** | **CCGGTAGTCGTCACGTCGAAATGAAG** |
| **Fwd_4_** | **Lectin-based** | **CCGGAAGCGGAGATGCTTGCCG** |
| **LecSF_1_** | **Lectin-based** | **TTGGTGCCTGCACGTGGAGT** |
| **LecSR_1_** | **Lectin-based** | **ACATTTTCAGGTTCAGACCCTTCTT** |
| **LecSF_2_** | **Lectin-based** | **AGGGCTGCCTGCTCGTGGAGG** |
| **LecSR_2_** | **Lectin-based** | **GACTTTTCATCGTACCCTTCCC** |
| **LecFLF** | **Lectin-based** | **GAATCTTTCAGTATGGAGTTATATGGACTG** |
| **LecFLR** | **Lectin-based** | **TAGTAATAAT TACATTTTCAGGTTCAG** |
